# PBRM1 Deficiency Sensitizes Renal Cancer Cells to DNMT Inhibitor 5-Fluoro-2’-Deoxycytidine

**DOI:** 10.3389/fonc.2022.870229

**Published:** 2022-06-03

**Authors:** Di Gu, Kai Dong, Aimin Jiang, Shaoqin Jiang, Zhibin Fu, Yewei Bao, Fuzhao Huang, Chenghua Yang, Linhui Wang

**Affiliations:** ^1^ Department of Urology, Changhai Hospital, Naval Medical University, Shanghai, China; ^2^ Department of Urology, Fujian Union Hospital, Fujian Medical University, Fuzhou, China

**Keywords:** PBRM1 gene mutation, DNA methyltransferase inhibitor, renal cell carcinoma, FdCyd, synthetic lethality

## Abstract

PBRM1 is a tumor suppressor frequently mutated in clear cell renal cell carcinoma. However, no effective targeted therapies exist for ccRCC with PBRM1 loss. To identify novel therapeutic approaches to targeting PBRM1-deficient renal cancers, we employed a synthetic lethality compound screening in isogenic PBRM1+/+ and PBRM1-/- 786-O renal tumor cells and found that a DNMT inhibitor 5-Fluoro-2’-deoxycytidine (Fdcyd) selectively inhibit PBRM1-deficient tumor growth. RCC cells lacking PBRM1 show enhanced DNA damage response, which leads to sensitivity to DNA toxic drugs. Fdcyd treatment not only induces DNA damage, but also re-activated a pro-apoptotic factor XAF1 and further promotes the genotoxic stress-induced PBRM1-deficient cell death. This study shows a novel synthetic lethality interaction between PBRM1 loss and Fdcyd treatment and indicates that DNMT inhibitor represents a novel strategy for treating ccRCC with PBRM1 loss-of-function mutations.

## Introduction

Worldwide, renal cell carcinoma (RCC) represents the sixth most frequently diagnosed cancer in men and the 10th in women, accounting for 5% and 3% of all oncological diagnoses, respectively (1). Clear cell renal cell carcinoma (ccRCC) is the most common histological subtype of kidney cancer and somatic inactivation of VHL occurs in about 70% of sporadic ccRCCs (2, 3). Although loss of VHL is the initiating event in ccRCC, the acquisition of additional mutations have to accumulate to finally give rise to ccRCC. Polybromo 1 (PBRM1), a tumor suppressor gene encoding the BAF180 protein, has been identified by next-generation sequencing as the second most frequently mutated genes in ccRCC (4). PBRM1 functions as a chromatin-targeting subunit of SWI/SNF chromatin remodeling complexes (5). Its mutation further activates the HIF response and cooperates with the VHL mutation to generate ccRCC in mouse models (6–8). The loss of PBRM1 expression was also associated with aggressive features and advanced stage, as well as with worse prognosis (9–11).

Since the fact of the high frequency of mutations and loss of expression of PBRM1 in tumor, one approach for developing new therapy options in ccRCC would be to identify targets that have synthetic lethal relationships with PBRM1 loss. Synthetic lethality is defined as the setting in which inactivation of either of two genes individually does not affect cell viability but loss of function of both genes simultaneously causes lethality. In cancer, this means identifying targeted therapies that cause selective lethality in the cancer cells that lack a specific tumor suppressor gene but spare normal cells (12). Recent studies have found that PBRM1 has a synthetic lethality interaction with genes involved in some epigenetic regulation, including EZH2 (13, 14) and poly ADP-ribose polymerase 1 (PARP1) (15). These researches suggested that PBRM1 mutant cancer cells will create dependence on other epigenetic machinery component to maintain cellular survival. With this hypothesis, we employed a druggable synthetic lethality screen using an epigenetic compound library and a PBRM1 isogenic ccRCC pair. Among the screen, we identified 5-Fluoro-2′-deoxycytidine (FdCyd), a DNA methyltransferase inhibitors (DNMTi) as synthetic lethality drugs for PBRM1-deficient ccRCC cells.

FdCyd is an antineoplastic agent that inhibits DNA methyltransferase and DNA methylation by acting as cytidine antimetabolite-forming covalent complexes with DNMTis and DNA (16). It is currently under assessment in clinical trials of various advanced solid tumors (17, 18). DNMTis induce global hypomethylation, which results in the re-expression of certain tumor suppressor genes (19, 20). On the other hand, the covalent trapping also causes DNA damage that was shown to be involved in the cytotoxic effects of DNMTi (20).

In this study, we describe that a DNA methyltransferase inhibitor FdCyd induces synthetic lethality in PBRM1-deficient ccRCC. Further mechanical studies reveal that FdCyd causes DNA damage and reactivates the tumor suppressor gene XAF1, leading to an activation of p53 signaling pathways, followed by cell cycle arrest at G2/m phase and apoptosis in PBRM1-deficient cells. Our data provide a strong evidence of novel PBRM1 synthetic lethality targets in human epigenetics modifiers.

## Materials and Methods

### Cell Lines and Culture

Human ccRCC cell lines 786-O, Caki-1 and HEK 293T cell line were obtained from from ATCC (Rockville, MD, USA). 786-O cells were cultured in RPMI-1640 media containing 10% FBS and 1% pen/strep. Caki-1 cells were cultured in McCoy’s 5A media containing 10% FBS and 1% pen/strep. HEK 293t cells were cultured in DMEM media containing 10% FBS and 1% pen/strep. All the cells were incubated in a humidified incubator adjusted with 5% CO2 at 37°C. All the cells were routinely screened for mycoplasma absence.

### Generation of PBRM1−/− Cells and Lentivirus Preparation

PBRM1 gene knockout was performed in786-O, Caki-1 cell lines using a CRISPR/Cas9-based gene editing technique. All sgRNAs were cloned into the lenti-CRISPR-V2 plasmids (Addgene plasmid #52961). Cells were transfected with the PBRM1 KO constructs using the lentiviral transduction. Then the transduced cells were selected by puromycin (Solarbio). Isolated KO clones were verified with Western blotting with and Sanger sequencing of the genomic PBRM1 locus. To produce lentiviruses, CRISPR-V2 plasmid, pMD2.G (Addgene plasmid # 12559) and psPAX2 (Addgene plasmid #12260) were co-transfected into HEK 293t cells using LentiFit (Hanbio). At 48 h post-transfection, cell supernatants were harvested and virus was concentrated using PEG-8000. The information of the primer sequences is available in **Supplementary Table 1**.

### Epigenetics Compound Library Screen and Cell Viability Measurement

Epigenetics Compound Library (L1200, 773 epigenetics compounds) was purchased from Topscience. After the the first round of screening, 107 compounds were prepared in 384-well plates with an eight-dose, interplate titration format, ranging from 14 nM to 30 μM of final concentrations. 786-O PBRM1+/+ or PBRM1−/− #1 cells were seeded at 800 cells per well in the 384-well plates containing the compound library and incubated at 37°C in a CO2 incubator for 72 h. All the liquid handling was done with Bravo Automated Liquid Handling Platform (Agilent). For cell viability measurement, cells were incubated with CellTiter-Glo^®^ 2.0 solution (Promega, USA) for 10 min and the fluorescence was measured with a SPECTROstar Nano Microplate Reader (BMG Labtech). The screen was done in duplicated and the half maximal inhibitory concentration (IC50) of each compound for the isogenic cell pair were calculated with GraphPad Prism 8. Synthetic lethality hits were calculated according to the following formula: SI = IC50PBRM1 (+/+)/IC50PBRM1(−/−). Drugs with SI > 2 were choosed as synthetic lethality candidates.

### RNA Isolation and RT-qPCR

Total RNA was harvested from cultured cells using Trizol reagent (Invitrogen, USA). RT was then performed with the PrimeScript™ RT reagent Kit with gDNA Eraser (Takara, Japan). qPCR was performed using TB Green^®^ Premix Ex Taq™ II (Takara, Japan). The sequences of primers used are included in **Supplementary Table 1**.

### Western Blot Analysis

Total protein from cells was harvested with ice-cold RIPA buffer (Thermo Scientific) plus protease inhibitor cocktail (Topscience). Proteins were fractionated by SDS–PAGE and transferred onto PVDF membrane (Bio-Rad). Membranes were incubated with the indicated primary antibodies overnight at 4°C and further incubated with the corresponding HRP-conjugated secondary antibodies (Epizyme, 1:5000) for 1.5 h at room temperature. Target proteins were detected using ECL Substrate (Epizyme) under an Amersham Imager 600 (GE Healthcare Life Sciences).

### Tumor Xenograft Mouse Model

All animal procedures were approved by the Institutional Animal Care and Use Committee at the PLA Naval Medical University and were undertaken in accordance with the NIH Guide for the Care and Use of Laboratory Animals. Five-weeks-old, female BALB/c nude mice were implanted in the left flanks with 786-O PBRM1+/+ and PBRM1−/− #1 cells suspended in Matrigel. When palpable tumors became detectable, animals with each tumor were divided randomly into 2 groups (n = 5 mice per group) for treatment with vehicle and Fdcyd. Mice were treated with vehicle (sterile saline containing 0.5% Hydroxy propyl methyl cellulose and 0.2% Tween80) or Fdcyd (25mg/kg, po, daily) for 21 days. The tumor sizes were measured by a Vernier caliper and calculated based on the modified ellipsoid formula (long axis × short axis2 × π/6). The mouse body weight was measured regularly to assess potential compound toxicity. At the end of experiments, mice were sacrificed and the tumors were harvested for weighing and further analyses.

### Cell Cycle and Apoptosis Assays

786-O PBRM1+/+ and PBRM1−/− cells grown in 6-well plates were treated with Fdcyd for 24 h. For cell cycle analysis, cells were harvested for propidium iodide (PI) staining. For apoptosis analysis, Annexin V-FITC Apoptosis Detection Kit (Beyotime) was used. The cell fluorescence was analyzed with a BD FACSCalibur flow cytometer and the data was analyzed by FlowJo software.

### Immunofluorescence Analyses

Cells were washed twice with PBS and fixed in 4% PFA for 15 min at room temperature. Cells were then treated with PBST (0.1% Triton X-100 in PBS), followed by blocking with 2% goat serum, 5% BSA, 0.5% Tween20 for 1 h. After that, cells were incubated with primary antibodies overnight at 4°C and further incubated with a secondary fluorescence-conjugated antibody for 60 min at room temperature in the dark. The nuclei were stained with DAPI Staining Solution (Abcam 228549).

### Bisulfite Modification and Methylation-Specific PCR

Genomic DNA (1 ug), harvested with a TIANamp Genomic DNA Kit (Tiangen), was used for bisulfite modification with the DNA Bisulfite Conversion Kit (Tiangen). Bisulfite modified DNA was used for methylation-specific PCR (MSP) reaction with designed primers. The information of the primer sequences is available in **Supplementary Table 1**.

### Statistical Analysis

All data were expressed as the mean ± standard deviation (s.d.). Statistical significance of differences between control and test groups was determined by Student’s t test or two-way ANOVA using Graphpad Prism 6 (GraphPad Software, La Jolla, CA). P values <0.05 were considered significant.

## Results

### Epigenetic Compound Library Screen Identifies DNMT Inhibitors as Synthetic Lethal Drugs in PBRM1-Deficient Renal Cancer Cells

**Figure 1:**
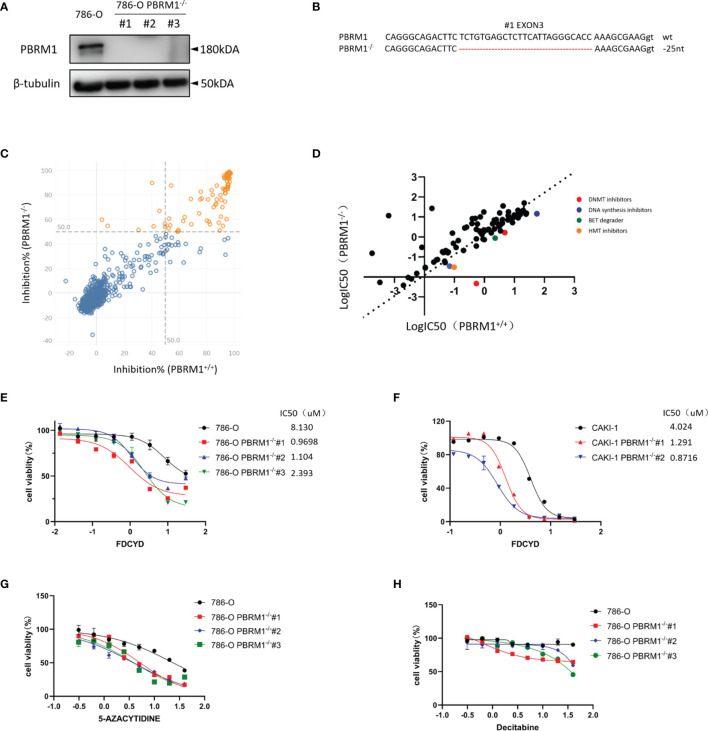
Epigenetic compound library screen identifies DNMT inhibitors as synthetic lethal drugs in PBRM1-deficient renal cancer cells. **(A)** Western Blot analysis showing loss of PBRM1 expression in the three PBRM1−/− clones. **(B)** The genomic Sanger sequencing of PBRM1 locus in 786-O PBRM1+/+ and PBRM1-/-(#1) cells. PBRM1−/− clone#1 lost 25 nucleotides in exon 3. **(C)** A compound inhibition rate plot of the first round screen data are shown. **(D)** A log10-IC50 plot of the second round screen data are shown. The IC50 values of the compounds against 786-O PBRM1+/+ and PBRM1-/- cells was plotted. Compounds with selectivity index (SI) > 2 for PBRM1−/− cells were chosen as synthetic lethality candidates. **(E–H)** Cell viability assay was done to certify the synthetic lethality effect by Fdcyd in 786-O isogenic pairs **(E)** and CAKI-1 isogenic pairs **(F)**. The other two DNMTis 5-Azacytidine **(G)** and Decitabine **(H)** were also used to test the IC50 in 786-O isogenic pairs. Error bars represent s.d. (n = 9) from three independent experiments. ANOVA P value of <0.001 for Fdcyd, 5-Azacytidine and Decitabine.

To screen and identify PBRM1 synthetic lethality targets, we first generate PBRM1 knockout (KO) 786-O cells with CRISPR-Cas9 gene editing system. The use of PBRM1 isogenic cell pairs will ensure that the identified effects operate in one particular genetic background. PBRM1 KO was verified with qPCR analysis (**Figure S1A**), Western blot and genomic Sanger sequencing of the sgRNA target regions with PBRM1 gene (**Figures 1A, B**). The growth rate of 786-O isogenic cell pairs were measured using CellTiter-Glo (**Figure S1B**). Among the three proved PBRM1 KO clones (PBRM1−/− #1–3), PBRM1−/− #1 was used for the synthetic lethality screen and the other clones were used for hits validation. To screen for clinical actionable vulnerabilities affiliated with PBRM1 deficiency, we used a library of 774 small-molecule targeting most of druggable human epigenetic proteins. The screen was done by two process. First, cells were exposed to epigenetic compounds of 10uM concentration for three days, after which, drug inhibition rate was assessed using CellTiter-Glo (**Figure 1C**). Next, drugs with inhibition rates greater than 50% were arrayed in 384-well plates with eight-dose to determine the estimated IC50 values for the PBRM1 isogenic pair (PBRM1+/+ and PBRM1−/− #1 cells) (**Figure 1D**). After two-rounds screen, we identified six candidate drugs that showed a selectivity index (SI) >2 for the PBRM1−/− #1 cells. We selected Fdcyd for further study as: (i) the best candidate synthetic lethality hit was Fdcyd; (ii) the parallel small molecule inhibitor screen contained two DNMTi (Fdcyd and 5-Azacytidine) as candidate synthetic lethal drugs; and (iii) Fdcyd is a DNA methyltransferase (DMNT) inhibitor currently used in solid tumor clinical trials. To verify the screening results, we examined Fdcyd in parental 786-O PBRM1+/+ and three PBRM1−/− clones (#1, #2, and #3). All three PBRM1−/− 786-O clones were significantly more sensitive to Fdcyd than the parental PBRM1+/+ cells (**Figure 1E**). Similar results were obtained with the CAKI1 PBRM1+/+ and two CAKI1−/− clones (**Figure 1F**). We also tested another DNMTis, 5-Azacytidine and Decitabine, in PBRM1-isogenic 786-O pair for synthetic lethality effect (**Figures 1G, H**). These results suggested that PBRM1-deficient RCC cells are synthetic lethal with the inhibitors of DNMT.

### Fdcyd Induces Apoptosis and G2/S Cell Cycle Arrest Selectively in PBRM1−/− RCC Cells

**Figure 2:**
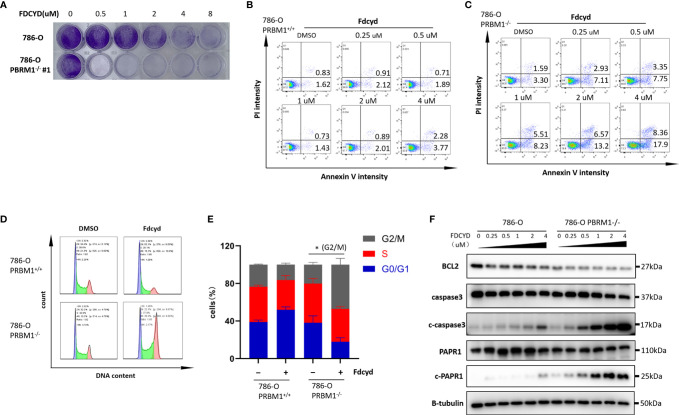
Fdcyd induces apoptosis and G2/S cell cycle arrest selectively in PBRM1−/− 786-O cells. **(A)** Colony formation assay of 786-O PBRM1+/+ and 786-O PBRM1−/− cells treated with indicated concentrations of Fdcyd for 14 days. **(B, C)** Flow cytometer analysis Annexin V/PI double stained 786-O PBRM1+/+ and 786-O PBRM1−/− cells treated with Fdcyd. **(D)** Flow cytometer analysis propidium iodide (PI)-stained 786-O PBRM1+/+ and 786-O PBRM1−/− cells treated with Fdcyd. **(E)** Percentage of cell populations in G1, S, and G2/M phase from the flow cytometry analysis. **(F)** Immunoblot analysis of BCL2, PARP1 and caspase-3. The cleavage of PARP1 and caspase-3 was shown as markers of apoptosis induction. Data are mean ± SD of three independent experiments. *P < 0.05, student’s t test.

To further explore the synthetic lethality of Fdcyd, we examined the effect of Fdcyd on the colony forming ability of PBRM1-isogenic 786-O cells and CAKI-1 cells. Treatment of PBRM1−/− RCC cells with Fdcyd for 7days impaired colony formation, whereas wild-type RCC cells were relatively resistant to Fdcyd treatment (**Figures 2A**, **S4A, B**). Next, to test whether the synthetic lethality effect accompanied cell apoptosis or cell cycle arrest, we first performed Annexin V/PI double staining and analyzed cells with flow cytometry. Fdcyd selectively induced apoptosis in PBRM1−/− RCC cells (**Figures 2B, C**, **S4C**). Similarly, treatment with 1 μM Fdcyd for 24h increased the percentage of cells in the G2M phase from 22% to 50% (**Figures 2D, E**, **S4D, E**). To further substantiate our conclusion, we analyzed the expression of the cleavage of PARP1/caspase-3 by western blot. These two apoptosis markers increased obviously in PBRM1-/- 786-O cells treated with Fdcyd (**Figures 2F**, **S4F**). Meanwhile, the anti-apoptosis factor BCL-2 was found down-regulated in PBRM1-/- 786-O cells comparing to PBRM1+/+ 786-O cells, especially after Fdcyd treatment. These results demonstrated that Fdcyd-induced synthetic lethality in PBRM1−/− RCC cells by inducing apoptosis and cell cycle arrest.

### Fdcyd Altered the Transcription of Genes Involved in P53 Pathway and DNA Damage Responses

**Figure 3:**
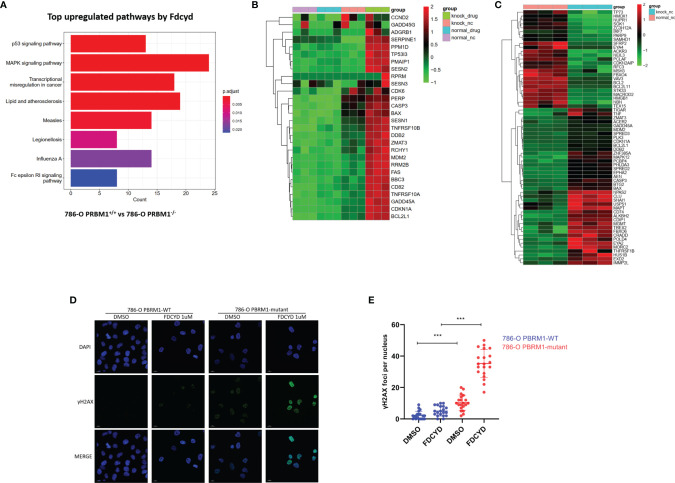
Fdcyd altered the transcription of genes involved in P53 pathway and DNA damage responses. **(A)** The KEGG pathway analysis was manipulated to identify the most significantly up-regulated pathways by Fdcyd in PBRM1-deficient 786-O cells. **(B, C)** Heatmap image of comparing gene expression profiles in p53 pathway and DNA damage is shown. Heatmap of Z scores(range:-2 to +2) displaying relative gene expression across all clusters. **(D)** Immunofluorescence staining of γH2AX foci in Fdcyd and DMSO treated 786-O PBRM1-isogenic cells. Scale bars, 10 µm. **(E)** Quantification of the number ofγH2AX foci per nuclei in 786-O PBRM1-isogenic cells treated with Fdcyd or DMSO. A minimum of 20 nuclei per condition were analyzed. Data are mean ± SD of three independent experiments. ***P < 0.001, student’s t test.

To explore molecular pathways underlying the synthetic lethality, we conducted transcriptome analysis of the PBRM1 isogenic 786-O cells with or without Fdcyd treatment. KEGG pathway analysis showed that gene sets related to p53 pathway were significantly enriched among genes up-regulated in PBRM1-deficient cells compared to 786-O control cells treated with Fdcyd (**Figures 3A, B**), suggesting that tumor cell death under Fdcyd treatment may due to the activation of p53 signaling pathway. On basis of this observation, we assumed that the cells treated with Fdcyd might evoke DNA damage responses. Indeed, several genes related to pressure response and DNA repair were up-regulated in PBRM1-deficient cells compared to 786-O control cells in the absence of exogenous DNA damage (**Figure 3C**), which means persisting DNA damage or impaired DNA damage repair (DDR) may accumulate in 786-O. PBRM1-deficient cells expressed higher levels of Pro-DNA damage response genes, including BAX, HUS1B, SREPD3, CASP3, EXD2, CLU, BTG2 and USP51. Additionally, gene expression of BCL2, PARP9, MACROD2, EYA4 and TEX15, which were involved in DNA repair and response to genotoxic stress was found down-regulated in PBRM1-deficient cells. Because sustained DNA damage usually leads to an increase in tumor mutation burden (TMB), we evaluated the tumor mutational burden (TMB) of PBRM1-defective ccRCC. Using whole-exome sequencing (WES) data from the Tumor Cancer Genome Atlas (TCGA), we found that PBRM1-mutant ccRCC had a significantly higher TMB than PBRM1-WT tumors (**, P < 0.01, Mann–Whitney U test, **Figure S6A**). Meanwhile, RCC data mining of GEPIA database demonstrated a positive correlation between expression of PBRM1 and DNA repair genes. (**Figures S6B–E**). To further test whether PBRM1-defective 786-O cells undergo severer DNA damage response, we monitored the presence of γH2AX and RAD51 in these cells treated with Fdcyd. We found an increase in the number of γH2AX and RAD51 foci after exposure to Fdcyd, an effect that was more pronounced in PBRM1−/− 786-O cells and CAKI-1 cells. (**Figures 3D, E**, **S4G–J**). Taken together, these results suggested that Fdcyd selectively induces DNA damage in PBRM1-deficient RCC cells.

### Reactivation of XAF1 by Fdcyd Was Associated With Demethylation of Hypermethylated Promoter

Since DNMTi can reactivate tumor suppressor gene through trapping of DNMT and inhibit the methylation mediated by enzymes during the following DNA replication (21), we speculated that the anti-tumor effect of Fdcyd was partial due to its demethylation process.

**Figure 4:**
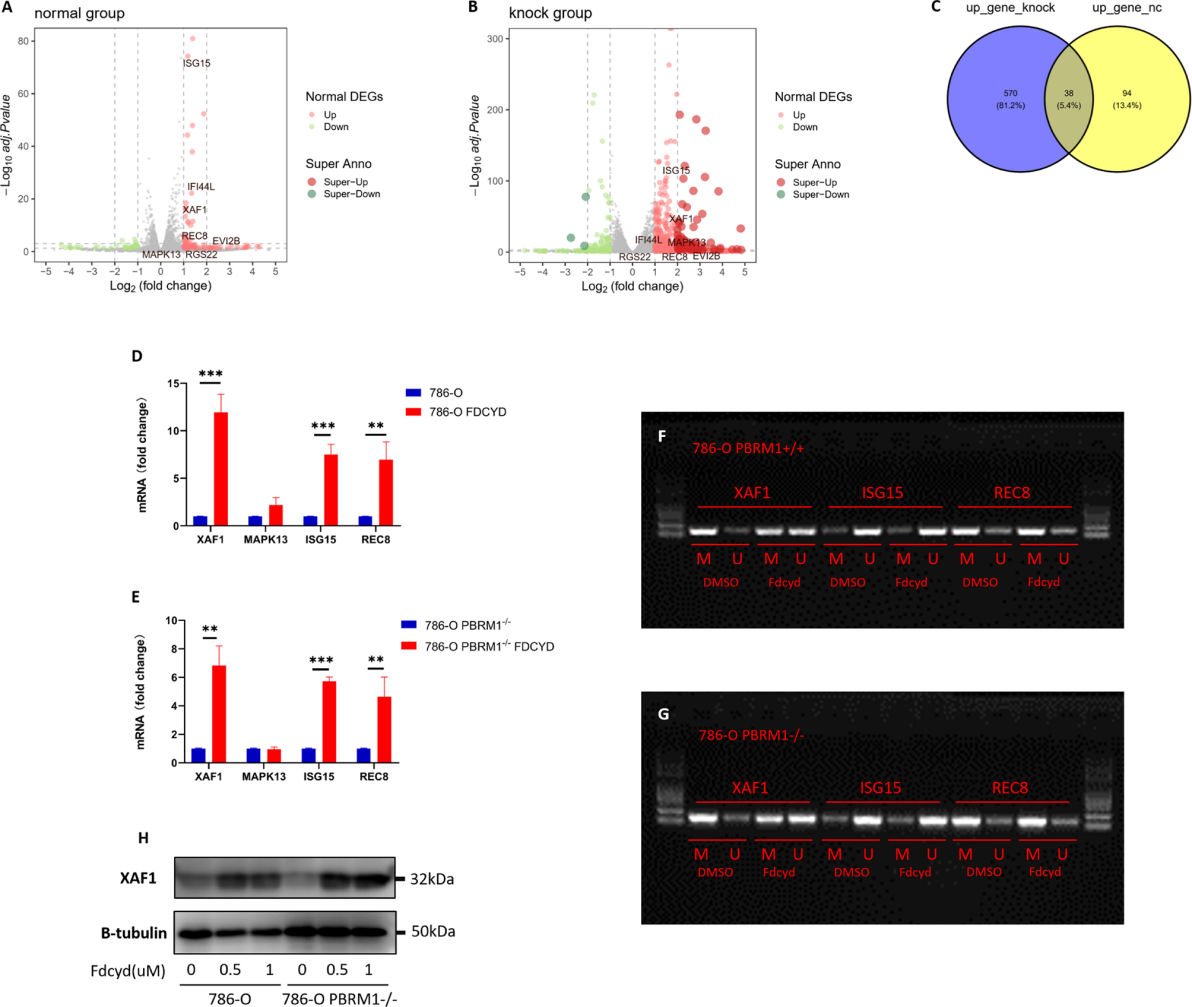
Reactivation of XAF1 by Fdcyd was associated with demethylation of hypermethylated promoter. **(A, B)** The volcano plot was conducted to identify differential expression of genes after Fdcyd treatment in 786-O isogenic pair. **(C)** The venn diagram showed gene sets that both up-regulated in Fdcyd treatment group. **(D, E)** Relative mRNA expression level of the indicated genes as determined by qPCR. Data are mean ± SD of three independent experiments. **P<0.01, ***P < 0.001, student’s t test. **(F, G)** Methylation-specific polymerase chain reaction confirmed demethylation of hypermethylated promoter region in 786-O isogenic cell pair. M, methylated DNA; U, unmethylated DNA. **(H)** Immunoblot analysis of XAF1. Data are mean ± SD of three independent experiments. **P<0.01***P < 0.001, ANOVA.

To define selection criteria for candidate tumor suppressor genes reactivated by Fdcyd, we first analyzed transcripts differentially regulated in 786-O PBRM1 isogenic pair with or without Fdcyd treatment. Thirty-eight genes were both up-regulated by Fdcyd (**Figures 4A–C**). Next, on the basis of the Tumor Suppressor Gene database (TSGene, https://bioinfo.uth.edu/TSGene/) (22), a set of seven candidate TSG genes (ISG15, XAF1, MAPK13, IFI44L, EVI2B, RGS22, REC8) was defined. Hypermethylation of CpG islands in the promoter regions of TSGs represents an important feature of DNA methylation aberrancies in cancer (23). As a third selection criterion for potential TSG genes, genes associated with a promoter hypermethylation phenotype were further investigated (**Figures S2A–D**). This reduced the number of potential genes to four candidates ISG15, XAF1, MAPK13 and REC8. QPCR confirmed that ISG15, XAF1 and REC8 mRNA were up-regulated in both isogenic cell lines (**Figures 4D, E**, **S5A**). To investigate whether increased gene expression was due to the reversal of promoter methylation, we performed methylation-specific polymerase chain reaction (MSP) and found that reactivation of XAF1 by Fdcyd was associated with demethylation of hypermethylated promoter (**Figures 4F, G**, **S5C**). Moreover, the protein expression of XAF1 was verified with western blots, showing that Fdcyd treatment significantly increased its level in both isogenic cell lines (**Figures 4H**, **S5B**). We also found increased protein expression of ISG15 in renal tumor cells after Fdcyd treatment (**Figure S2E**), although it may not due to the demethylation mechanism. XAF1 (XIAP Associated Factor 1) functions as a tumor suppressor by mediating apoptosis stress response of cancer cells (24, 25). In TCGA (The Cancer Genome Atlas) ccRCC data, patients with low expression of XAF1 have significantly worse prognosis than those expressing high levels (**Figure S2F**).

### Demethylation by Fdcyd of the Promoter of Tumor Suppressor Gene XAF1 Promotes Cell Apoptosis

**Figure 5:**
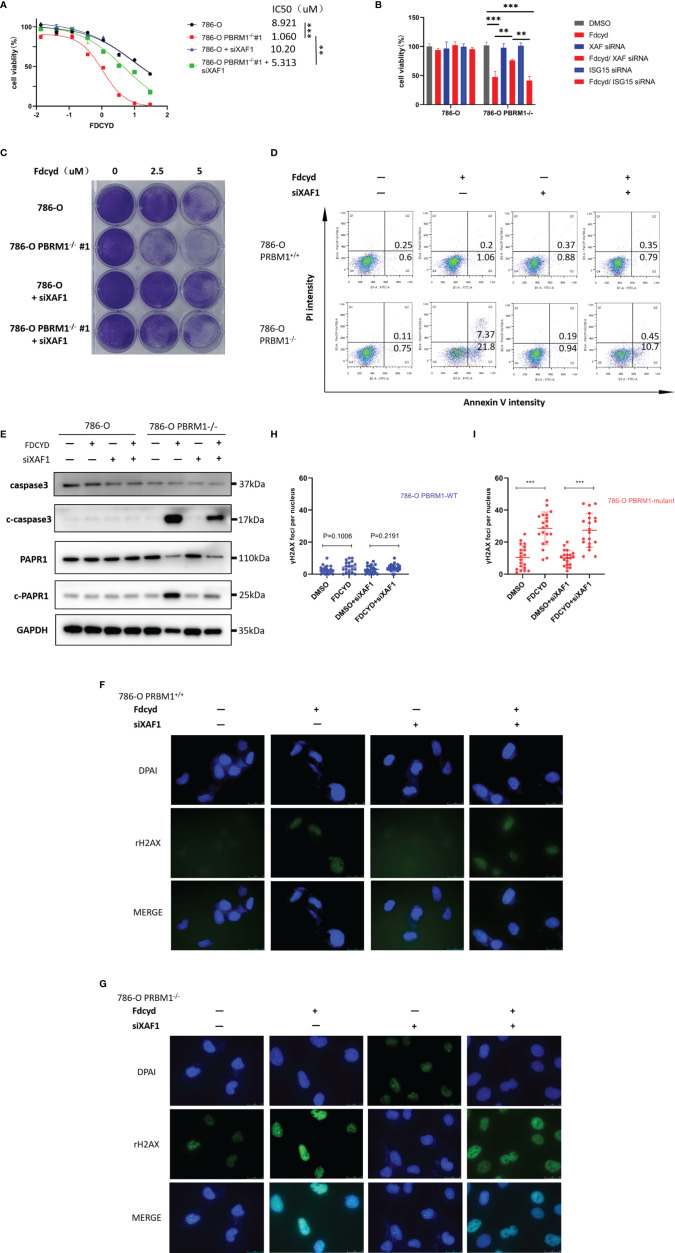
Demethylation by Fdcyd of the promoter of tumor suppressor gene XAF1 promotes cell apoptosis. **(A, B)** Effect of Fdcyd on cell viability and XAF1 siRNA rescue. 786-O isogenic cell pair were treated with 1uM Fdcyd with or without siRNA for 72h. Data are mean ± SD of three independent experiments. **P<0.01***P < 0.001, ANOVA. **(C)** Colony formation assay of 786-O PBRM1+/+ and 786-O PBRM1−/− cells treated with indicated concentrations of Fdcyd and XAF1 siRNA for 14 days. **(D)** Flow cytometer analysis Annexin V/PI double stained 786-O PBRM1+/+ and 786-O PBRM1−/− cells treated with Fdcyd and XAF1 siRNA. **(E)** Immunoblot analysis of PARP1 and caspase-3. The cleavage of PARP1 and caspase-3 was shown as markers of apoptosis induction. **(F, G)** Immunofluorescence staining of γH2AX foci in Fdcyd and XAF1 siRNA treated 786-O PBRM1-isogenic cells. Scale bars, 25 µm. **(H, I)** Quantification of the number of γH2AX foci per nuclei in 786-O PBRM1-isogenic cells treated with Fdcyd and XAF1 siRNA. A minimum of 20 nuclei per condition were analyzed. Data are mean ± SD of three independent experiments. ***P < 0.001, student’s t test.

To determine whether silencing of XAF1 conferred resistance to Fdcyd-induced cell death, 786-O isogenic pair cells were treated concurrently with Fdcyd and XAF1 siRNA. Fdcyd selectively inhibited the viability of PBRM1-deficient 786-O cells and this effect was significantly reversed by siRNA silencing of XAF1 gene (**Figures 5A, B**). Morevoer, the colony forming assay also showed that silencing of XAF1 did impair the growth inhibition effect of Fdcyd on PBRM1-deficient 786-O cells (**Figure 5C**). Because XAF1 has been implicated as playing a role in apoptosis pathways, we detected the apoptosis in Fdcyd and XAF siRNA co-treated cells. Results showed that silencing of XAF1 rescued the apoptosis induced by Fdcyd on PBRM1-deficient 786-O cells (**Figure 5D**). XAF siRNA reduced Fdcyd-induced apoptosis of PBRM1-deficient 786-O cells from 21.8% to 10.7%. This outcome was consistent with the western blot analysis of the expression of the cleavage of PARP1/caspase-3. XAF siRNA also reduced the cleavage of PARP1/caspase-3 expression induced by Fdcyd in PBRM1-deficient 786-O cells (**Figure 5E**). However, silencing of XAF1 had no effect on cell cycle arrest (**Figures S3A–C**). To further explore the role of XAF1 in Fdcyd-induced DNA damage, we performed γH2AX and RAD51 staining in these cells treated with Fdcyd and XAF1 siRNA. The number of γH2AX and RAD51 foci increased remarkably in PBRM1-deficient cells after exposure to Fdcyd (**Figures 5F–I**, **S3D–G**). Meanwhile, XAF1 siRNA did not seem to alleviate any DNA damage in PBRM1-deficient 786-O cells. Collectively, these data indicated that re-expression of gene XAF1 contributed to the cell cytotoxic effect of Fdcyd through apoptosis induction.

### Fdcyd Treatment Induces Synthetic Lethality in PBRM1-Deficient Renal Cancer *in vivo*

**Figure 6:**
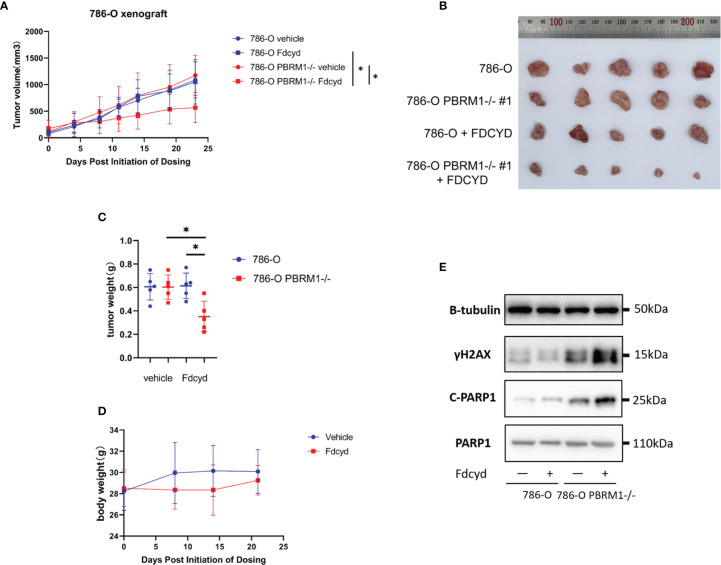
Fdcyd treatment induces synthetic lethality in PBRM1-deficient renal cancer in vivo. **(A)** Mice bearing 786-O or 786-O PBRM1-/- were treated daily with vehicle or Fdcyd with 25 mg/kg using oral gavage throughout the indicated treatment period. Data are mean ± SD. P=0.0111 between 786-O PBRM1-/- vehicle and Fdcyd treatment groups (n = 5), ANOVA. P=0.0251 between 786-O Fdcyd and 786-O PBRM1-/-Fdcyd treatment groups (n = 5), ANOVA. **(B)** Images of renal tumors excised from tumor-bearing mice. **(C)** Tumor weights were measured after harvested from the mice. Data are mean ± SD of tumor weights from four mice group (n = 5). *P < 0.05, Mann-Whitney U. **(D)** Body weight analysis of mice bearing tumor xenografts. **(E)** Immunoblot analysis illustrating the c-PARP1 and γH2AX protein levels in 786-O PBRM1+/+ and PBRM1−/− tumor tissues. .

To assess synthetic lethality by Fdcyd treatment *in vivo*, we evaluated the antitumor effect of Fdcyd in mice bearing established xenografts derived from PBRM1 isogenic cell pair. Mice bearing PBRM1 isogenic tumor xenografts were given vehicle or Fdcyd (25mg/kg, PO) daily for 21 days and tumor growth rate was monitored periodically. Compared with the vehicle group, Fdcyd treatment caused a delay in tumor growth of PBRM1-deficient 786-O xenografts, as measured by tumor volume (**Figures 6A, B**) and tumor weight (**Figure 6C**). Meanwhile, we did not see obvious weight loss in Fdcyd treated mice, suggesting the tolerable toxicity of the drug (**Figure 6D**). WB analyses of tumor tissues revealed that Fdcyd treatment significantly induced γH2AX levels, as well as PARP1 cleavage in PBRM1-deficient 786-O tumors (**Figure 6E**). Together, our data demonstrated that Fdcyd treatment induced synthetic lethality in PBRM1-deficient renal cancer *in vivo*.

## Discussion

Mutations in genes encoding subunits of SWI/SNF chromatin-remodeling complexes are frequently observed in a large variety of human cancers, generally occurring in approximately 25% of all cancers (26). PBRM1, a component of the SWI/SNF complex, contains six acetyl-lysine binding bromodomains (BDs) and regulates gene expression including interferon stimulated gene factor and HIF related genes (27–29). Recently, PBRM1-defecient tumor cells were shown to be sensitized to PARP1 inhibitor (15). PBRM1-defective tumor cells exhibited elevated levels of DNA damage response and PARP inhibitor exposure exacerbated this phenotype.

Here, we conducted an epigenetic compound library screen and identified DNMTis as synthetic lethal compounds in PBRM1-deficient ccRCC cells. All three DNMTis Fdcyd, 5-Azacytidine and Decitabine from the library were found to be synthetic lethal in the test. The synthetic lethality of PBRM1 was verified in two different PBRM1 isogenic ccRCC pairs. Both 5-Azacytidine and Decitabine were FDA approved for the treatment of myelodysplastic syndrome (MDS) in 2004 and 2006 respectively (30, 31). Fdcyd is also currently under assessment in clinical trials of various advanced solid tumors, suggesting the finding might have translational utility in kidney cancer types which PBRM1 is mutated. These agents serve as cytidine antimetabolite-forming covalent complexes with DNMTs and DNA (32). DNMTs trapped in this form ultimately loss their function, leading to down-regulation of DNA methylation (33). This covalent trapping, however, also induces DNA damage that was suggested to be involved in the cytotoxic effects of DNMTis. Therefore, it might be reasoned that compounds with DNMTi activity generally induce supernormal toxicity in cells under DNA repair defect or stress response.

The transcriptional profile of the 786-O cells mutated in PBRM1 revealed a dramatic change in expression of genes responsible for DNA damage response (**Figure 3C**). These genes involved in double-strand breaks resection, homologous recombination and replicative stress response express higher levels in PBRM1-deficient 786-O and CAKI-1 cells without exogenous damage stimulus. Furthermore, nuclear γH2AX and RAD51 foci measurement showed higher preexisting levels of γH2AX and RAD51 foci in PBRM1-/- cells than in PBRM1+/+ cells. These results suggested that loss of PBRM1 gene function impairs the DNA damage response. Therefore, it can be postulated that PBRM1-defecient RCC cells have persistent DNA damage activation and may be sensitive to DNA damage drugs. Phenotypically, Fdcyd treatment induced significant DNA damage in PBRM1-deficient cells (**Figures 3D, E**). Increased DNA damage cause cellular G2/M arrest and apoptosis in PBRM1-deficient cells. Nonetheless, negligible DNA damage and apoptosis were observed in PBRM1-WT cells upon Fdcyd treatment. This phenotype is in accordance with previous observations that Fdcyd treatment inhibited HCT116 cells at G2/M check point, induced apoptosis and amplified the DNA damage repair signal (34).

Abnormal hypermethylation of DNA may occur in renal cell carcinoma (RCC), resulting in tumor suppressor genes silenced and tumorigenesis (35). Many TSGs have been reported to be partially or completely silenced because of the hypermethylation of their promoter regions leading to drug resistance (36). We wondered whether reactivation of silenced TSGs by Fdcyd might also contribute to the synthetic lethal effect. To answer this question, we found three reactivated tumor suppressor genes XAF1, ISG15 and REC8 in Fdcyd treated 786-O cells. MSP analysis further confirmed that hypermethylation of a CpG Island in the promoter of XAF1 was found in 786-O and CAKI-1 cells and Fdcyd treatment led to XAF1 promoter region demethylation (**Figure 4F**). This was associated with re-activation of XAF1 mRNA and protein expression (**Figure 4H**). The X-linked inhibitor of apoptosis (XIAP)-associated factor 1 (XAF1) was involved in apoptosis signaling by antagonizing the anti-apoptotic activity of XIAP and the anti-Caspase activity (37, 38). It was also reported that XAF1 promotes the genotoxic stress-induced cell death response through different activities (39). To further confirm the role of XAF1 in Fdcyd treatment on tumor cells, we assessed whether re-expression of XAF1 could alleviate apoptosis, DNA damage and cell cycle arrest. The results reflect limited effect of XAF1 on DNA damage and cell cycle arrest. However, inhibition of XAF1 by siRNA in Fdcyd treated 786-O cells considerably alleviated apoptosis (**Figure 5D**). These observations suggested that XAF1 played an important role in apoptosis induction by Fdcyd. These data suggested a direct correlation between XAF1 re-expression and Fdcyd demethylation.

Recently, inactivation of PRBM1 was found to sensitized tumor cells to T cell–mediated killing and increased

Type I interferon response (40). Interestingly, an association between PBRM1 loss of function, present in ~60% of ccRCC, and response to immunotherapy has been reported, though the association has not been observed consistently (41–44). On the other side, anti-tumor DNA-demethylation agents were reported to upregulate immune signaling and interferon response pathway (45, 46). Investigating whether DNA-demethylation agents may have clinical relevance for immune modulation in PBRM1-deficient renal tumor cells remains an important question that warrants further studies.

In summary, results of our *in vitro* and mouse xenograft *in vivo* studies indicated that Fdcyd treatment was synthetic lethal with PBRM1 loss in ccRCC and Fdcyd could serve as a novel therapeutic agent for renal cancer with PBRM1 deficiency.

## Data Availability Statement

The datasets presented in this study can be found in online repositories. The names of the repository/repositories and accession number(s) can be found in the article/**Supplementary Material**.

## Ethics Statement

The animal study was reviewed and approved by the Institutional Animal Care and Use Committee at the PLA Naval Medical University.

## Author Contributions

LW and CY contributed to conception and design of the study. DG and KD performed all of the experiments with assistance from AJ, YB, SJ and FH. DG wrote the paper and prepared diagrams. KD and ZF participated in the material preparation and manuscript review. All authors have read and approved the manuscript for publication.

## Funding

This study was funded by the National Natural Science Foundation of China (no. 81730073 and 81872074 to LW).

## Conflict of Interest

The authors declare that the research was conducted in the absence of any commercial or financial relationships that could be construed as a potential conflict of interest.

## Publisher’s Note

All claims expressed in this article are solely those of the authors and do not necessarily represent those of their affiliated organizations, or those of the publisher, the editors and the reviewers. Any product that may be evaluated in this article, or claim that may be made by its manufacturer, is not guaranteed or endorsed by the publisher.

## Supplementary Material

Supplementary Figure 1
**(A)** Relative mRNA expression level of PBRM1 as determined by qPCR. Data are mean ± SD of three independent experiments. **(B)** The growth rate analysis of 786-O isogenic cell pairs.

Supplementary Figure 2
**(A–D)** CpG island prediction of XAF1, MAPK13, ISG15 and REC8. **(E)** Western Blot analysis showing increased XAF1 expression after Fdcyd treatment in 786-O PBRM1+/+ and 786-O PBRM1−/− cells. **(F)** Survivorship curve for PFS according to XAF1 expression from TCGA data base.

Supplementary Figure 3
**(A)** Flow cytometer analysis propidium iodide (PI)-stained 786-O PBRM1+/+ and 786-O PBRM1−/− cells treated with Fdcyd and XAF1 siRNA. **(B, C)** Percentage of cell populations in G1, S, and G2/M phase from the flow cytometry analysis. **(D, E)** Immunofluorescence staining of RAD51 foci in Fdcyd and XAF1 siRNA treated 786-O PBRM1-isogenic cells. Scale bars, 25 µm. **(F, G)** Quantification of the number of RAD51 foci per nuclei in 786-O PBRM1-isogenic cells treated with Fdcyd and XAF1 siRNA. A minimum of 20 nuclei per condition were analyzed. Data are mean ± SD of three independent experiments. ***P < 0.001, student’s t test.

Supplementary Figure 4
**(A, B)** Colony formation assay of CAKI-1 PBRM1+/+ and CAKI-1 PBRM1−/− cells treated with indicated concentrations of Fdcyd for 14 days. **(C)** Flow cytometer analysis Annexin V/PI double stained CAKI-1 PBRM1+/+ and CAKI-1 PBRM1−/− cells treated with Fdcyd. **(D)** Flow cytometer analysis propidium iodide (PI)-stained CAKI-1 PBRM1+/+ and CAKI-1 PBRM1−/− cells treated with Fdcyd. **(E)** Percentage of cell populations in G1, S, and G2/M phase from the flow cytometry analysis. Data are mean ± SD of three independent experiments. *P < 0.05, student’s t test. **(F)** Immunoblot analysis of PARP1 and caspase-3. The cleavage of PARP1 and caspase-3 was shown as markers of apoptosis induction. **(G, H)** Immunofluorescence staining of γH2ax and RAD51 foci in Fdcyd treated CAKI-1 PBRM1-isogenic cells. Scale bars, 25 µm. **(I, J)** Quantification of the number of γH2ax and RAD51 foci per nuclei in CAKI-1 PBRM1-isogenic cells treated with Fdcyd. A minimum of 20 nuclei per condition were analyzed. Data are mean ± SD of three independent experiments. ***P < 0.001, student’s t test.

Supplementary Figure 5
**(A)** Relative mRNA expression level of gene XAF1 as determined by qPCR. Data are mean ± SD of three independent experiments. **P<0.01, student’s t test. **(B)** Immunoblot analysis of XAF1. **(C)** Methylation-specific polymerase chain reaction confirmed demethylation of hypermethylated promoter region in CAKI-1 isogenic cell pair. **(D, E)** Persistence of γH2AX foci over time in PBRM1-mutant 786-O cells after exposure to Fdcyd. **(F)** Immunoblot analysis of PBRM1 knockout efficiency in CAKI-1 cells. **(G)** Immunoblot analysis of Knockdown efficiency of siXAF1 in 786-O cells. **(H)** Relative mRNA expression level of the indicated genes as determined by qPCR. Data are mean ± SD of three independent experiments. **P<0.01, student’s t test. **(I, J)** Immunoblot analysis illustrating the c-PARP1 and γH2AX protein levels in 786-O PBRM1+/+ and PBRM1−/− tumor tissues.

Supplementary Figure 6
**(A)** Comparison of TMB between PBRM1-WT and PBRM1-mutant renal cell carcinomas in TCGA database. PBRM1-mutant ccRCC had a significantly higher TMB than PBRM1-WT tumors (**, P < 0.01,Mann–Whitney U test). **(B–E)** Correlation between mRNA expression of PBRM1 and DNA repair genes in the GEPIA database. **(F)** GSEA analysis of p53 signaling pathway between PBRM1-deficient cells compared to 786-O control cells treated with Fdcyd.

## References

[1] Siegel RL Miller KD Jemal A. Cancer Statistics, 2018. CA Cancer J Clin (2018) 68:7–30. doi: 10.3322/caac.21442 29313949

[2] Moch H Cubilla AL Humphrey PA Reuter VE Ulbright TM. The 2016 WHO Classification of Tumours of the Urinary System and Male Genital Organs-Part A: Renal, Penile, and Testicular Tumours. Eur Urol (2016) 70:93–105. doi: 10.1016/j.eururo.2016.02.029 26935559

[3] Gnarra JR Tory K Weng Y Schmidt L Wei MH Li H. Mutations of the VHL Tumour Suppressor Gene in Renal Carcinoma. Nat Genet (1994) 7:85–90. doi: 10.1038/ng0594-85 7915601

[4] Varela I Tarpey P Raine K Huang D Ong CK Stephens P. Exome Sequencing Identifies Frequent Mutation of the SWI/SNF Complex Gene PBRM1 in Renal Carcinoma. Nature (2011) 469:539–42. doi: 10.1038/nature09639 PMC3030920 21248752

[5] Mashtalir N D'Avino AR Michel BC Luo J Pan J Otto JE. Modular Organization and Assembly of SWI/SNF Family Chromatin Remodeling Complexes. Cell (2018) 175:1272–88.e20. doi: 10.1016/j.cell.2018.09.032 30343899 PMC6791824

[6] Gao W Li W Xiao T Liu XS Kaelin WG Jr. Inactivation of the PBRM1 Tumor Suppressor Gene Amplifies the HIF-Response in VHL-/- Clear Cell Renal Carcinoma. Proc Natl Acad Sci USA (2017) 114:1027–32. doi: 10.1073/pnas.1619726114 PMC5293026 28082722

[7] Gu YF Cohn S Christie A McKenzie T Wolff N Do QN. Modeling Renal Cell Carcinoma in Mice: Bap1 and Pbrm1 Inactivation Drive Tumor Grade. Cancer Discovery (2017) 7:900–17. doi: 10.1158/2159-8290.CD-17-0292 PMC5540776 28473526

[8] Nargund AM Pham CG Dong Y Wang PI Osmangeyoglu HU Xie Y. The SWI/SNF Protein PBRM1 Restrains VHL-Loss-Driven Clear Cell Renal Cell Carcinoma. Cell Rep (2017) 18:2893–906. doi: 10.1016/j.celrep.2017.02.074 PMC5431084 28329682

[9] Pawlowski R Muhl SM Sulser T Krek W Moch H Schraml P. Loss of PBRM1 Expression is Associated With Renal Cell Carcinoma Progression. Int J Cancer (2013) 132:E11–7. doi: 10.1002/ijc.27822 22949125

[10] da Costa WH Rezende M Carneiro FC Rocha RM da Cunha IW Carraro DM. Polybromo-1 (PBRM1), a SWI/SNF Complex Subunit is a Prognostic Marker in Clear Cell Renal Cell Carcinoma. BJU Int (2014) 113:E157–63. doi: 10.1111/bju.12426 24053427

[11] Carril-Ajuria L Santos M Roldán-Romero JM Rodriguez-Antona C de Velasco G. Prognostic and Predictive Value of PBRM1 in Clear Cell Renal Cell Carcinoma. Cancers (2019) 12:16. doi: 10.3390/cancers12010016 PMC7016957 31861590

[12] Huang A Garraway LA Ashworth A Weber B. Synthetic Lethality as an Engine for Cancer Drug Target Discovery. Nat Rev Drug Discovery (2020) 19:23–38. doi: 10.1038/s41573-019-0046-z 31712683

[13] Kim KH Kim W Howard TP Vazquez F Tsherniak A Wu JN. SWI/SNF-Mutant Cancers Depend on Catalytic and non-Catalytic Activity of EZH2. Nat Med (2015) 21:1491–6. doi: 10.1038/nm.3968 PMC4886303 26552009

[14] Huang K Sun R Chen J Yang Q Wang Y Zhang Y. A Novel EZH2 Inhibitor Induces Synthetic Lethality and Apoptosis in PBRM1-Deficient Cancer Cells. Cell Cycle (2020) 19:758–71. doi: 10.1080/15384101.2020.1729450 PMC7145336 32093567

[15] Chabanon RM Morel D Eychenne T Colmet-Daage L Bajrami I Dorvault N. PBRM1 Deficiency Confers Synthetic Lethality to DNA Repair Inhibitors in Cancer. Cancer Res (2021) 81:2888–902. doi: 10.1158/0008-5472.CAN-21-0628 33888468

[16] Smith SS Kaplan BE Sowers LC Newman EM. Mechanism of Human Methyl-Directed DNA Methyltransferase and the Fidelity of Cytosine Methylation. Proc Natl Acad Sci USA (1992) 89:4744–8. doi: 10.1073/pnas.89.10.4744 PMC49160 1584813

[17] Holleran JL Beumer JH McCormick DL Johnson WD Newman EM Doroshow JH. Oral and Intravenous Pharmacokinetics of 5-Fluoro-2'-Deoxycytidine and THU in Cynomolgus Monkeys and Humans. Cancer Chemother Pharmacol (2015) 76:803–11. doi: 10.1007/s00280-015-2857-x PMC4573928 26321472

[18] Coyne GO Wang L Zlott J Juwara L Covey JM Beumer JH. Intravenous 5-Fluoro-2'-Deoxycytidine Administered With Tetrahydrouridine Increases the Proportion of P16-Expressing Circulating Tumor Cells in Patients With Advanced Solid Tumors. Cancer Chemother Pharmacol (2020) 85:979–93. doi: 10.1007/s00280-020-04073-5 PMC7188725 32314030

[19] Qin T Si J Raynal NJ Wang X Gharibyan V Ahmed S. Epigenetic Synergy Between Decitabine and Platinum Derivatives. Clin Epigenetics (2015) 7:97. doi: 10.1186/s13148-015-0131-z 26366234 PMC4567801

[20] Chovanec M Taza F Kalra M Hahn N Nephew KP Spinella MJ. Incorporating DNA Methyltransferase Inhibitors (DNMTis) in the Treatment of Genitourinary Malignancies: A Systematic Review. Target Oncol (2018) 13:49–60. doi: 10.1007/s11523-017-0546-x 29230671 PMC6428576

[21] Mehdipour P Murphy T De Carvalho DD. The Role of DNA-Demethylating Agents in Cancer Therapy. Pharmacol Ther (2020) 205:107416. doi: 10.1016/j.pharmthera.2019.107416 31626871

[22] Zhao M Kim P Mitra R Zhao J Zhao Z. TSGene 2.0: An Updated Literature-Based Knowledgebase for Tumor Suppressor Genes. Nucleic Acids Res (2016) 44:D1023–31. doi: 10.1093/nar/gkv1268 PMC4702895 26590405

[23] Dizman N Philip EJ Pal SK. Genomic Profiling in Renal Cell Carcinoma. Nat Rev Nephrol (2020) 16:435–51. doi: 10.1038/s41581-020-0301-x 32561872

[24] Pinto EM Figueiredo BC Chen W Galvao HCR Formiga MN Fragoso M. XAF1 as a Modifier of P53 Function and Cancer Susceptibility. Sci Adv (2020) 6:eaba3231. doi: 10.1126/sciadv.aba3231 32637605 PMC7314530

[25] Jeong SI Kim JW Ko KP Ryu BK Lee MG Kim HJ. XAF1 Forms a Positive Feedback Loop With IRF-1 to Drive Apoptotic Stress Response and Suppress Tumorigenesis. Cell Death Dis (2018) 9:806. doi: 10.1038/s41419-018-0867-4 30042418 PMC6057933

[26] Mittal P Roberts CWM. The SWI/SNF Complex in Cancer - Biology, Biomarkers and Therapy. Nat Rev Clin Oncol (2020) 17:435–48. doi: 10.1038/s41571-020-0357-3 PMC8723792 32303701

[27] Cai W Su L Liao L Liu ZZ Langbein L Dulaimi E. PBRM1 Acts as a P53 Lysine-Acetylation Reader to Suppress Renal Tumor Growth. Nat Commun (2019) 10:5800. doi: 10.1038/s41467-019-13608-1 31863007 PMC6925188

[28] Gao YH Wu ZX Xie LQ Li CX Mao YQ Duan YT. VHL Deficiency Augments Anthracycline Sensitivity of Clear Cell Renal Cell Carcinomas by Down-Regulating ALDH2. Nat Commun (2017) 8:15337. doi: 10.1038/ncomms15337 28643803 PMC5481740

[29] Liao L Liu ZZ Langbein L Cai W Cho EA Na J. Multiple Tumor Suppressors Regulate a HIF-Dependent Negative Feedback Loop *via* ISGF3 in Human Clear Cell Renal Cancer. Elife (2018) 7:e37925. doi: 10.7554/eLife.37925 30355451 PMC6234029

[30] Kaminskas E Farrell AT Wang YC Sridhara R Pazdur R. FDA Drug Approval Summary: Azacitidine (5-Azacytidine, Vidaza) for Injectable Suspension. Oncologist (2005) 10:176–82. doi: 10.1634/theoncologist.10-3-176 15793220

[31] Fenaux P. Inhibitors of DNA Methylation: Beyond Myelodysplastic Syndromes. Nat Clin Pract Oncol (2005) 2 Suppl 1:S36–44. doi: 10.1038/ncponc0351 16341239

[32] Venturelli S Berger A Weiland T Essmann F Waibel M Nuebling T. Differential Induction of Apoptosis and Senescence by the DNA Methyltransferase Inhibitors 5-Azacytidine and 5-Aza-2'-Deoxycytidine in Solid Tumor Cells. Mol Cancer Ther (2013) 12:2226–36. doi: 10.1158/1535-7163.MCT-13-0137 23924947

[33] Weisenberger DJ Velicescu M Cheng JC Gonzales FA Liang G Jones PA. Role of the DNA Methyltransferase Variant DNMT3b3 in DNA Methylation. Mol Cancer Res MCR. (2004) 2:62–72. doi: 10.1158/1541-7786.62.2.1 14757847

[34] Zhao Q Fan J Hong W Li L Wu M. Inhibition of Cancer Cell Proliferation by 5-Fluoro-2'-Deoxycytidine, a DNA Methylation Inhibitor, Through Activation of DNA Damage Response Pathway. SpringerPlus (2012) 1:65. doi: 10.1186/2193-1801-1-65 23397046 PMC3565089

[35] Avissar-Whiting M Koestler DC Houseman EA Christensen BC Kelsey KT Marsit CJ. Polycomb Group Genes are Targets of Aberrant DNA Methylation in Renal Cell Carcinoma. Epigenetics (2011) 6:703–9. doi: 10.4161/epi.6.6.16158 PMC3230543 21610323

[36] Liu A Wu Q Peng D Ares I Anadón A Lopez-Torres B. A Novel Strategy for the Diagnosis, Prognosis, Treatment, and Chemoresistance of Hepatocellular Carcinoma: DNA Methylation. Med Res Rev (2020) 40:1973–2018. doi: 10.1002/med.21696 32525219

[37] Liston P Fong WG Kelly NL Toji S Miyazaki T Conte D. Identification of XAF1 as an Antagonist of XIAP Anti-Caspase Activity. Nat Cell Biol (2001) 3:128–33. doi: 10.1038/35055027 11175744

[38] Fong WG Liston P Rajcan-Separovic E St Jean M Craig C Korneluk RG. Expression and Genetic Analysis of XIAP-Associated Factor 1 (XAF1) in Cancer Cell Lines. Genomics (2000) 70:113–22. doi: 10.1006/geno.2000.6364 11087668

[39] Matt S Hofmann TG. The DNA Damage-Induced Cell Death Response: A Roadmap to Kill Cancer Cells. Cell Mol Life Sci CMLS. (2016) 73:2829–50. doi: 10.1007/s00018-016-2130-4 PMC11108532 26791483

[40] Pan D Kobayashi A Jiang P Ferrari de Andrade L Tay RE Luoma AM. A Major Chromatin Regulator Determines Resistance of Tumor Cells to T Cell-Mediated Killing. Science (2018) 359:770–5. doi: 10.1126/science.aao1710 PMC5953516 29301958

[41] McDermott DF Huseni MA Atkins MB Motzer RJ Rini BI Escudier B. Clinical Activity and Molecular Correlates of Response to Atezolizumab Alone or in Combination With Bevacizumab Versus Sunitinib in Renal Cell Carcinoma. Nat Med (2018) 24:749–57. doi: 10.1038/s41591-018-0053-3 PMC6721896 29867230

[42] Braun DA Ishii Y Walsh AM Van Allen EM Wu CJ Shukla SA. Clinical Validation of PBRM1 Alterations as a Marker of Immune Checkpoint Inhibitor Response in Renal Cell Carcinoma. JAMA Oncol (2019) 5:1631–3. doi: 10.1001/jamaoncol.2019.3158 PMC6735411 31486842

[43] Motzer RJ Banchereau R Hamidi H Powles T McDermott D Atkins MB. Molecular Subsets in Renal Cancer Determine Outcome to Checkpoint and Angiogenesis Blockade. Cancer Cell (2020) 38:803–17.e4. doi: 10.1016/j.ccell.2020.10.011 33157048 PMC8436590

[44] Abou Alaiwi S Nassar AH Xie W Bakouny Z Berchuck JE Braun DA. Mammalian SWI/SNF Complex Genomic Alterations and Immune Checkpoint Blockade in Solid Tumors. Cancer Immunol Res (2020) 8(8):1075–84. doi: 10.1158/2326-6066.CIR-19-0866 PMC7415546 32321774

[45] Chiappinelli KB Strissel PL Desrichard A Li H Henke C Akman B. Inhibiting DNA Methylation Causes an Interferon Response in Cancer *via* dsRNA Including Endogenous Retroviruses. Cell (2015) 162:974–86. doi: 10.1016/j.cell.2015.07.011 PMC4556003 26317466

[46] Roulois D Loo Yau H Singhania R Wang Y Danesh A Shen Shu Y. DNA-Demethylating Agents Target Colorectal Cancer Cells by Inducing Viral Mimicry by Endogenous Transcripts. Cell (2015) 162:961–73. doi: 10.1016/j.cell.2015.07.056 PMC4843502 26317465

