# Supplementary figures and images for: PBRM1 Deficiency Sensitizes Renal Cancer Cells to DNMT Inhibitor 5-Fluoro-2’-Deoxycytidine
Source: Front Oncol. 2022 Jun 3;12:870229. doi: 10.3389/fonc.2022.870229 (PMC9204009; doi:10.3389/fonc.2022.870229)

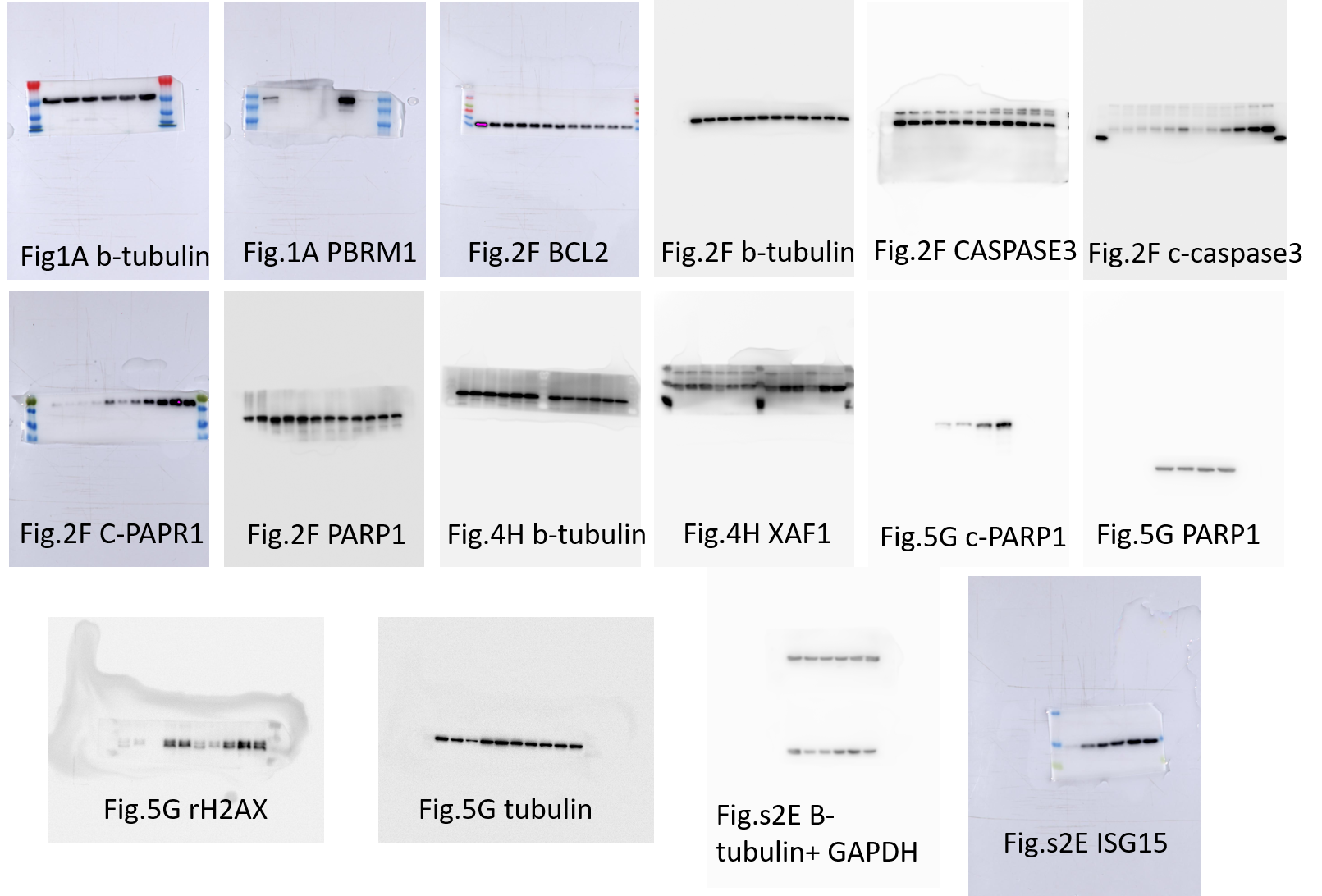

Supplement: Supplementary Figure 1 — (A) Relative mRNA expression level of PBRM1 as determined by qPCR. Data are mean ± SD of three independent experiments. (B) The growth rate analysis of 786-O isogenic cell pairs. [file Image_1.tif]

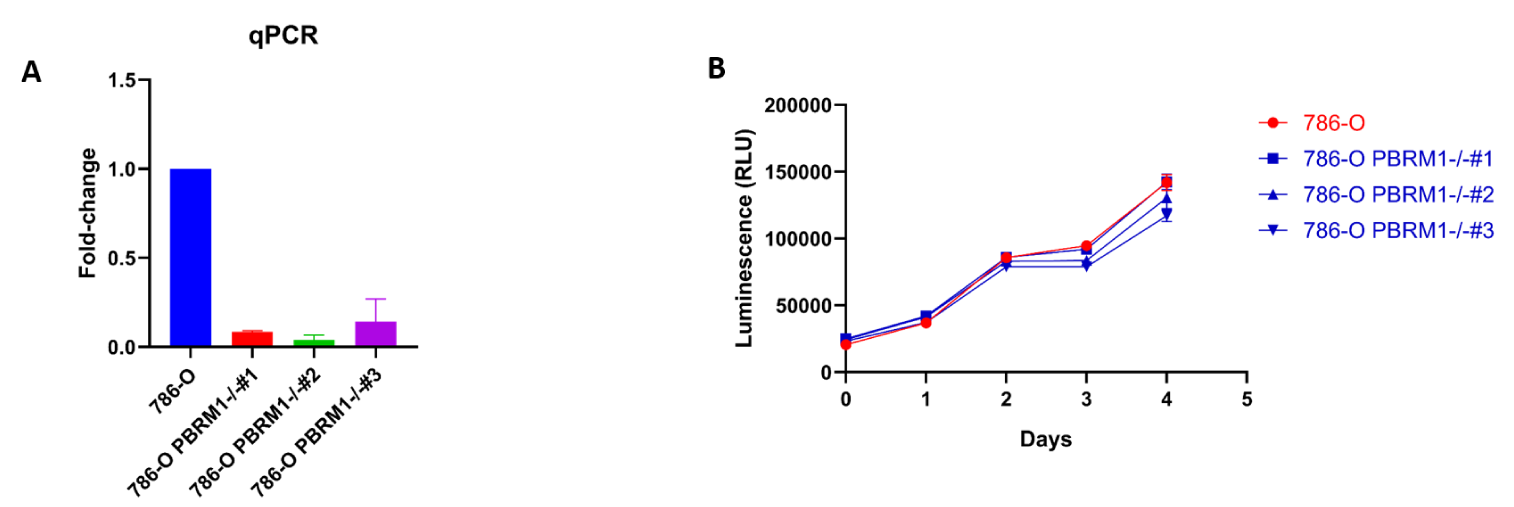

Supplement: Supplementary Figure 2 — (A–D) CpG island prediction of XAF1, MAPK13, ISG15 and REC8. (E) Western Blot analysis showing increased XAF1 expression after Fdcyd treatment in 786-O PBRM1+/+ and 786-O PBRM1−/− cells. (F) Survivorship curve for PFS according to XAF1 expression from TCGA data base. [file Image_2.tif]

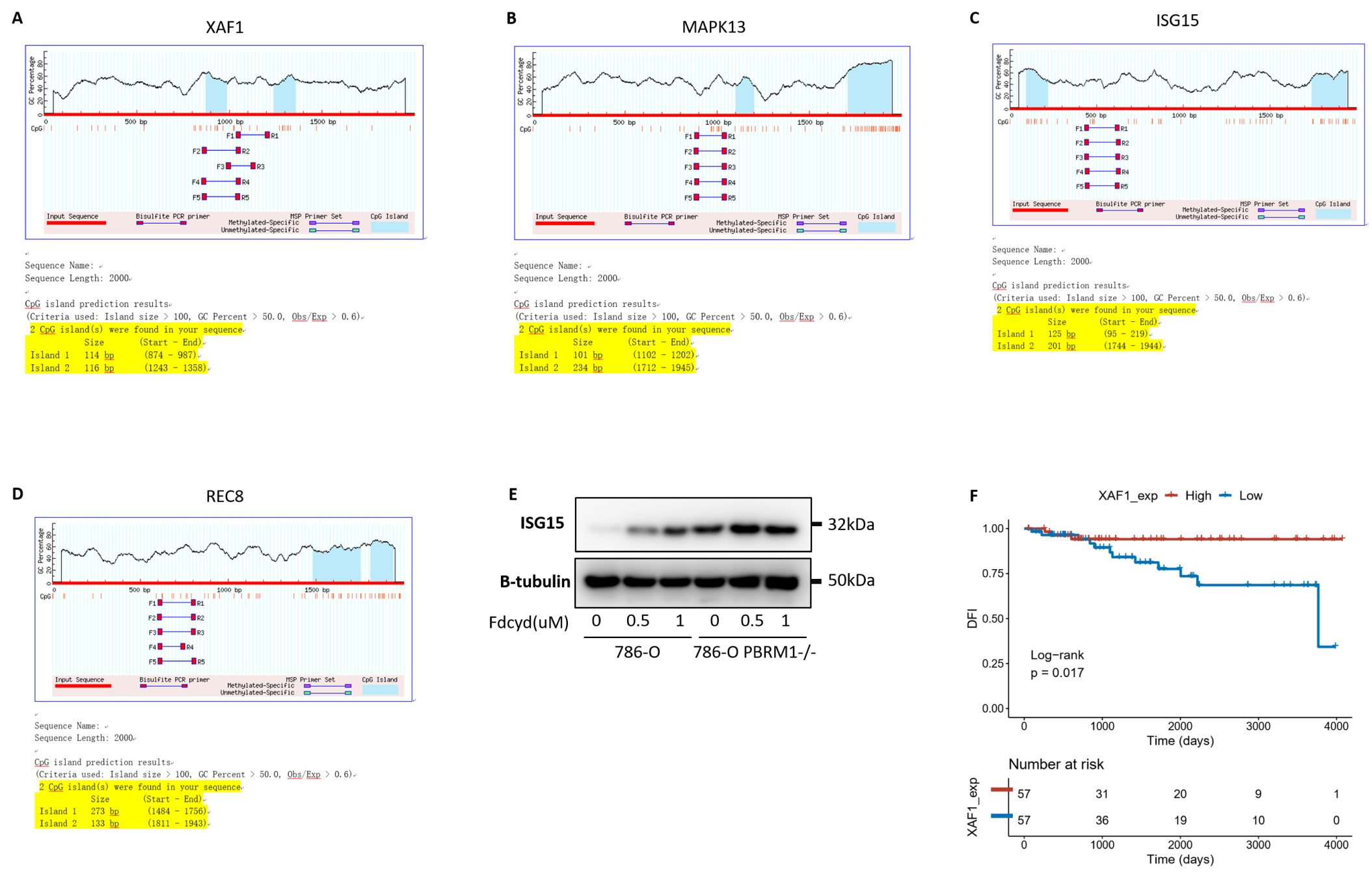

Supplement: Supplementary Figure 3 — (A) Flow cytometer analysis propidium iodide (PI)-stained 786-O PBRM1+/+ and 786-O PBRM1−/− cells treated with Fdcyd and XAF1 siRNA. (B, C) Percentage of cell populations in G1, S, and G2/M phase from the flow cytometry analysis. (D, E) Immunofluorescence staining of RAD51 foci in Fdcyd and XAF1 siRNA treated 786-O PBRM1-isogenic cells. Scale bars, 25 µm. (F, G) Quantification of the number of RAD51 foci per nuclei in 786-O PBRM1-isogenic cells treated with Fdcyd and XAF1 siRNA. A minimum of 20 nuclei per condition were analyzed. Data are mean ± SD of three independent experiments. ***P < 0.001, student’s t test. [file Image_3.tif]

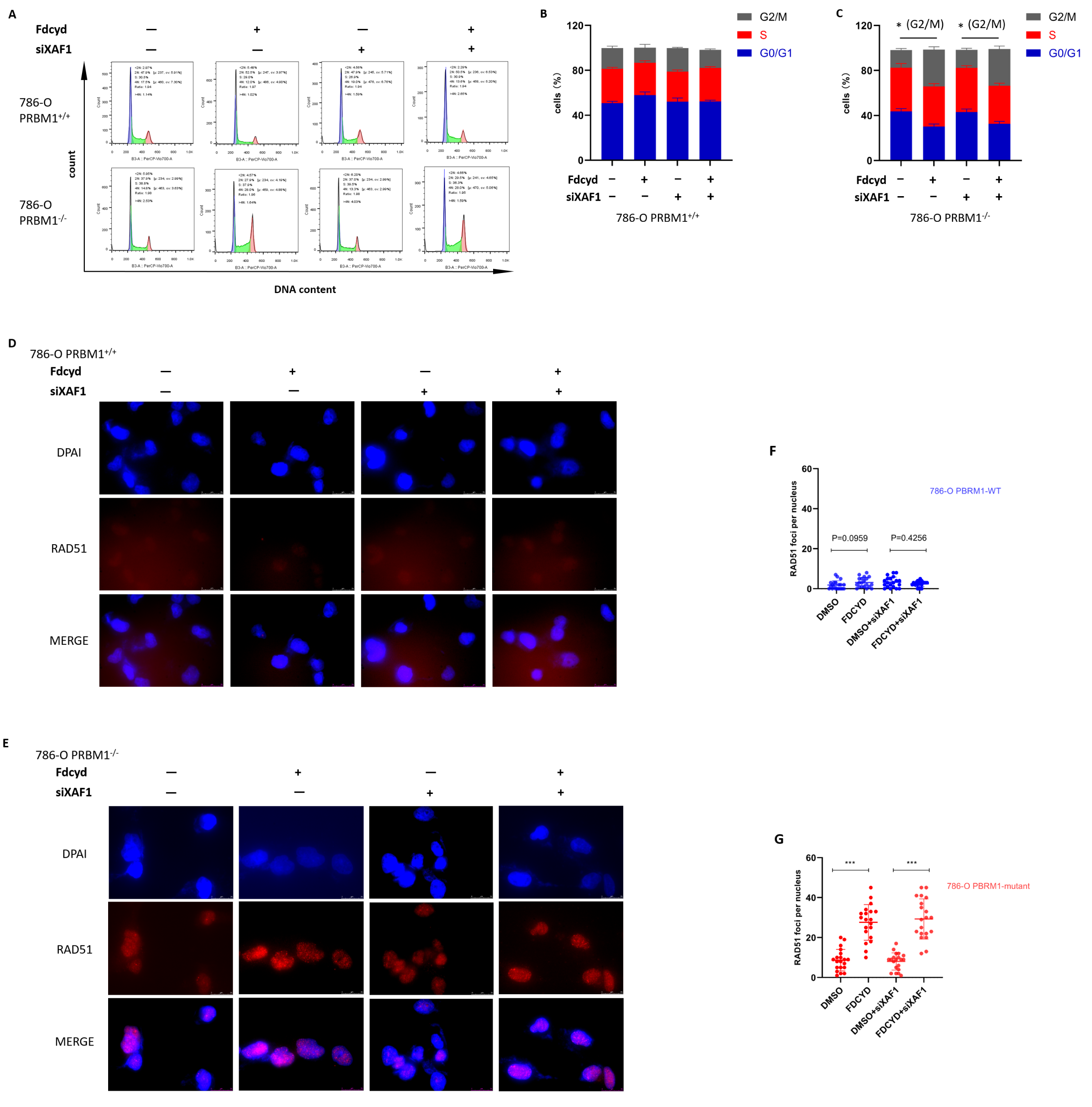

Supplement: Supplementary Figure 4 — (A, B) Colony formation assay of CAKI-1 PBRM1+/+ and CAKI-1 PBRM1−/− cells treated with indicated concentrations of Fdcyd for 14 days. (C) Flow cytometer analysis Annexin V/PI double stained CAKI-1 PBRM1+/+ and CAKI-1 PBRM1−/− cells treated with Fdcyd. (D) Flow cytometer analysis propidium iodide (PI)-stained CAKI-1 PBRM1+/+ and CAKI-1 PBRM1−/− cells treated with Fdcyd. (E) Percentage of cell populations in G1, S, and G2/M phase from the flow cytometry analysis. Data are mean ± SD of three independent experiments. *P < 0.05, student’s t test. (F) Immunoblot analysis of PARP1 and caspase-3. The cleavage of PARP1 and caspase-3 was shown as markers of apoptosis induction. (G, H) Immunofluorescence staining of γH2ax and RAD51 foci in Fdcyd treated CAKI-1 PBRM1-isogenic cells. Scale bars, 25 µm. (I, J) Quantification of the number of γH2ax and RAD51 foci per nuclei in CAKI-1 PBRM1-isogenic cells treated with Fdcyd. A minimum of 20 nuclei per condition were analyzed. Data are mean ± SD of three independent experiments. ***P < 0.001, student’s t test. [file Image_4.tif]

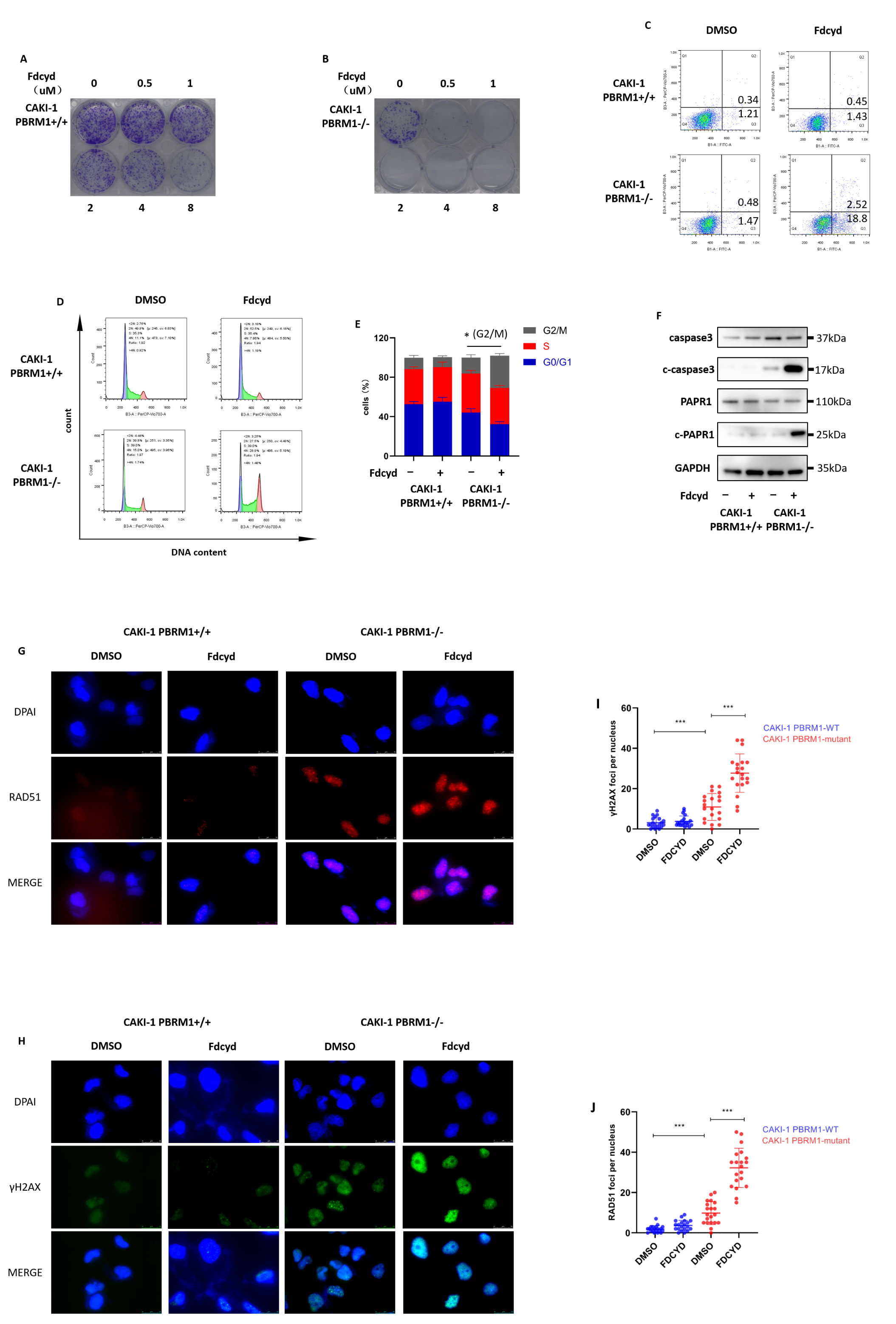

Supplement: Supplementary Figure 5 — (A) Relative mRNA expression level of gene XAF1 as determined by qPCR. Data are mean ± SD of three independent experiments. **P<0.01, student’s t test. (B) Immunoblot analysis of XAF1. (C) Methylation-specific polymerase chain reaction confirmed demethylation of hypermethylated promoter region in CAKI-1 isogenic cell pair. (D, E) Persistence of γH2AX foci over time in PBRM1-mutant 786-O cells after exposure to Fdcyd. (F) Immunoblot analysis of PBRM1 knockout efficiency in CAKI-1 cells. (G) Immunoblot analysis of Knockdown efficiency of siXAF1 in 786-O cells. (H) Relative mRNA expression level of the indicated genes as determined by qPCR. Data are mean ± SD of three independent experiments. **P<0.01, student’s t test. (I, J) Immunoblot analysis illustrating the c-PARP1 and γH2AX protein levels in 786-O PBRM1+/+ and PBRM1−/− tumor tissues. [file Image_5.tif]

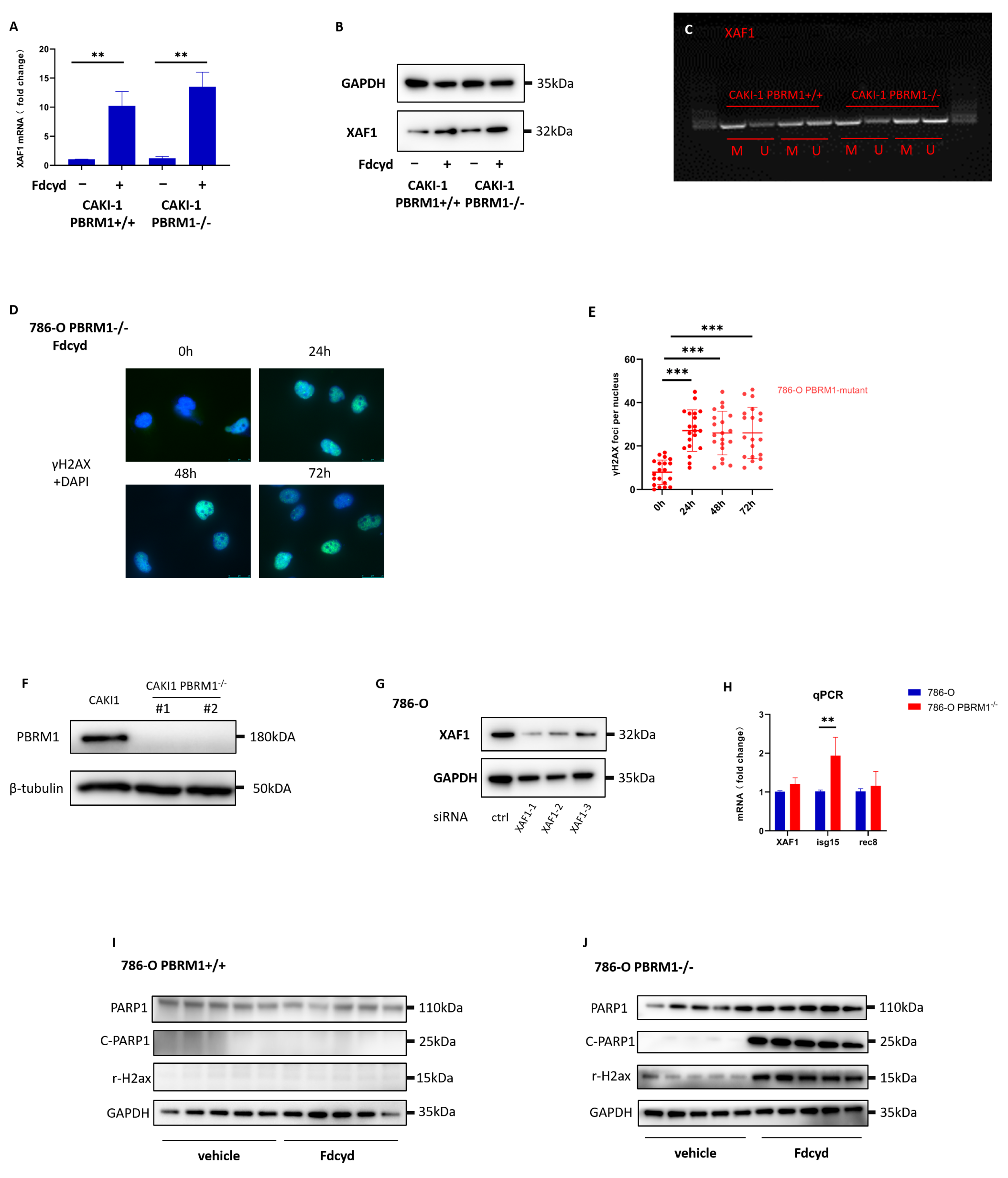

Supplement: Supplementary Figure 6 — (A) Comparison of TMB between PBRM1-WT and PBRM1-mutant renal cell carcinomas in TCGA database. PBRM1-mutant ccRCC had a significantly higher TMB than PBRM1-WT tumors (**, P < 0.01,Mann–Whitney U test). (B–E) Correlation between mRNA expression of PBRM1 and DNA repair genes in the GEPIA database. (F) GSEA analysis of p53 signaling pathway between PBRM1-deficient cells compared to 786-O control cells treated with Fdcyd. [file Image_6.tif]

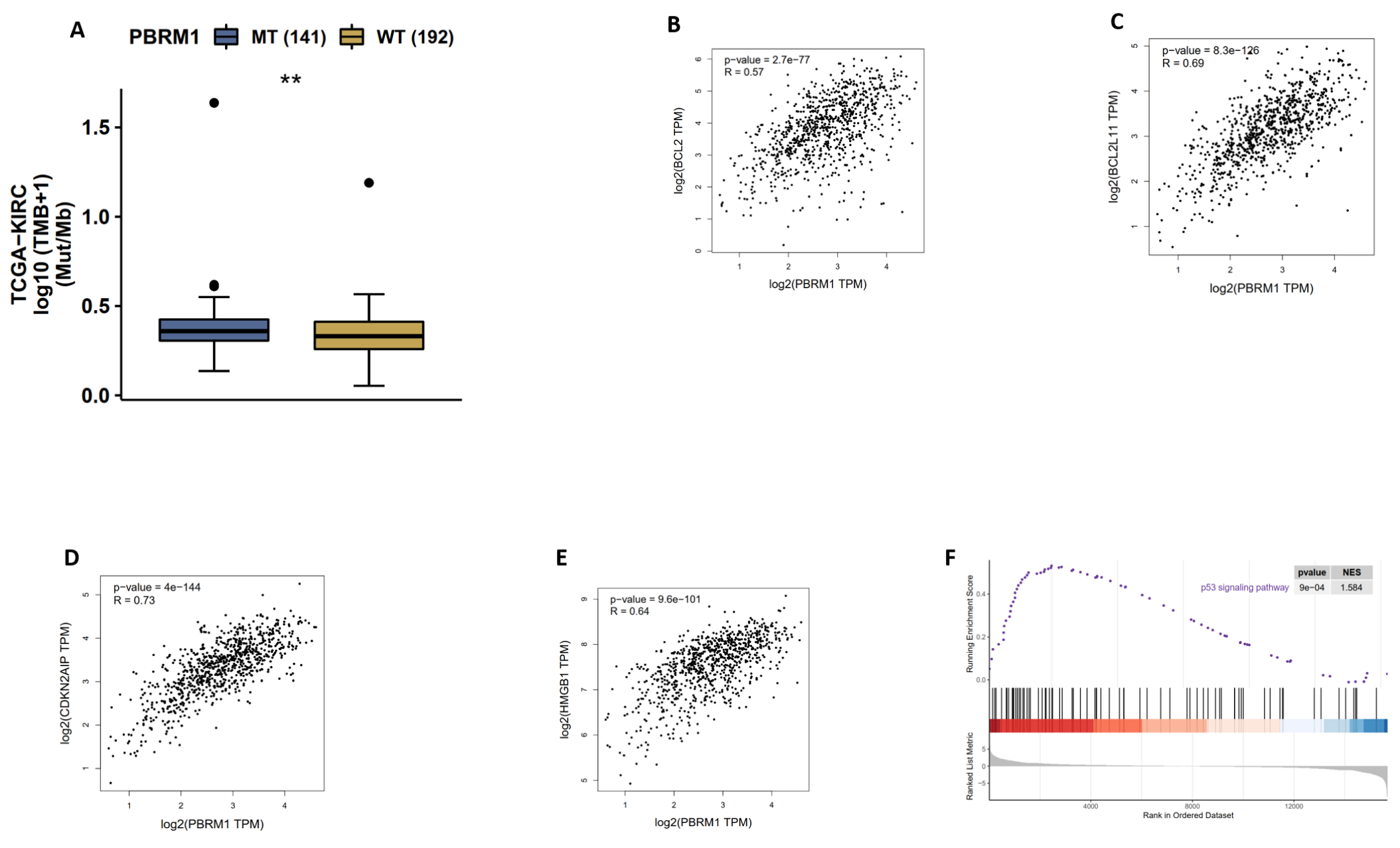

Supplement: Supplementary file 7 [file Image_7.tif]

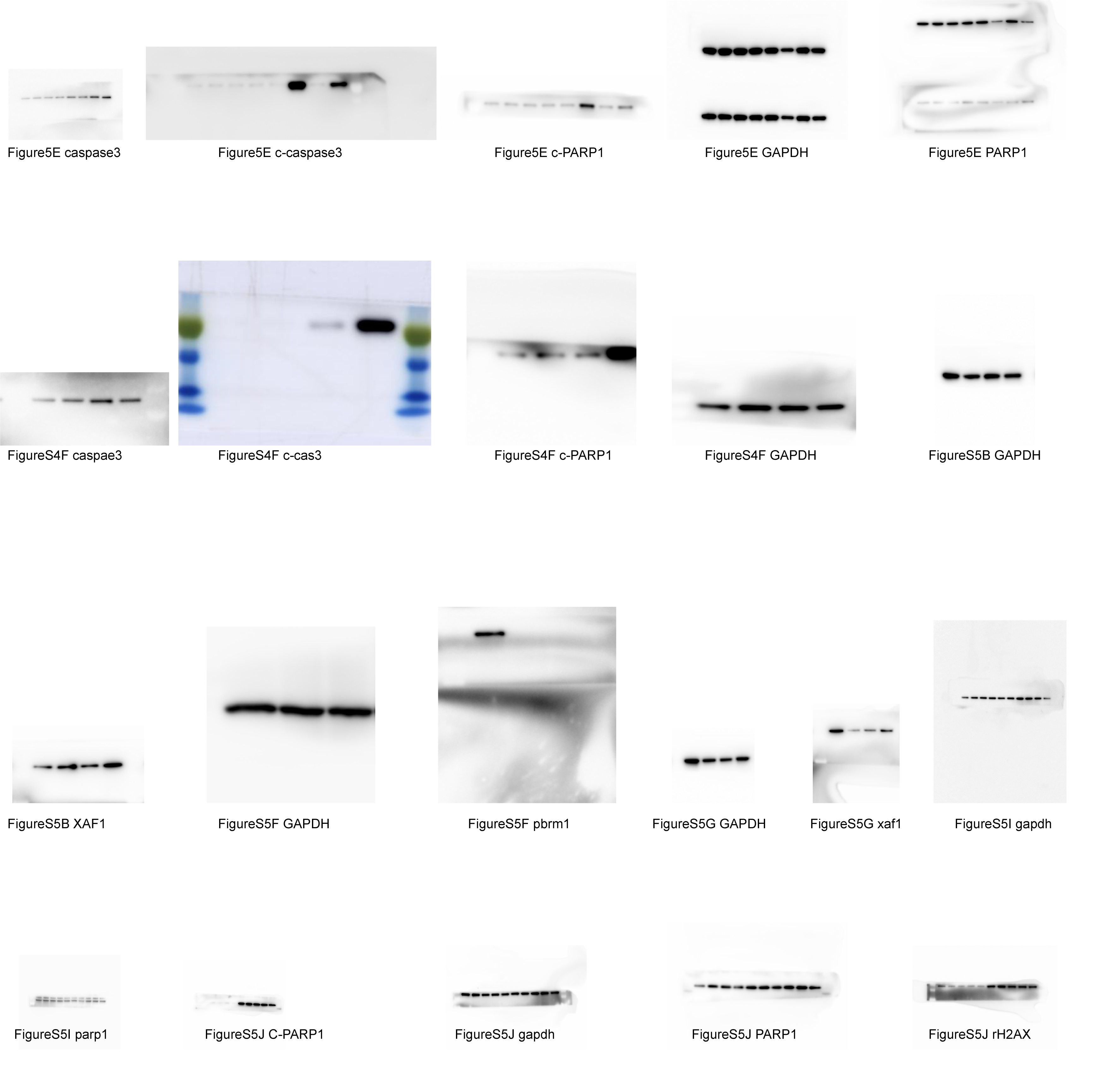

Supplement: Supplementary file 8 [file Image_8.tif]
