# Supplementary material for: PBRM1 Deficiency Sensitizes Renal Cancer Cells to DNMT Inhibitor 5-Fluoro-2’-Deoxycytidine
Source: Front Oncol. 2022 Jun 3;12:870229. doi: 10.3389/fonc.2022.870229 (PMC9204009; doi:10.3389/fonc.2022.870229)
Supplement: Supplementary file 9 [file Table_1.docx]

| Primer | Sequence |
| --- | --- |
| sgPBRM1-1 | CTCTGTGAGCTCTTCATTA |
| sgPBRM1-2 | TGGCAACCTGGTTCACCAT |
| sgPBRM1-3 | CGAGGAGATCTATATCTT |
| PBRM1 Forward | AGGAGGAGACTTTCCAATCTTCC |
| PBRM1 Reverse | CTTCGCTTTGGTGCCCTAATG |
| XAF1 Forward | ATGGAAGGAGACTTCTCGGT |
| XAF1 Reverse | TTGCTGAGCTGCATGTCCAG |
| ISG15 Forward | CTCTGAGCATCCTGGTGAGGAA |
| ISG15 Reverse | AAGGTCAGCCAGAACAGGTCGT |
| REC8 Forward | GTTGTTCAGAACCCCAACTC |
| REC8 Reverse | AAGACACCATAAGGGGAACA |
| MAPK13 Forward | GAGAAGGTGGCCATCAAGAA |
| MAPK13 Reverse | GTCCTCATTCACAGCCAGGT |
| XAF1 (methylated) Forward | GTTTGTAAGAAACGAAATTTAATCGA |
| XAF1 (methylated) Reverse | GCCAACCCGAATCTACCG |
| XAF1 (unmethylated) Forward | TGTTTGTAAGAAATGAAATTTAATTGAAAG |
| XAF1 (unmethylated) Reverse | TCACCAACCCAAATCTACCAC |
| ISG15 (methylated) Forward | CGTTCGTTGTTCGGAGTTTTC |
| ISG15 (methylated) Reverse | AAACGTACCGCCGAACCTAC |
| ISG15 (unmethylated) Forward | TGTTGTTTGTTGTTTGGAGTTTTT |
| ISG15 (unmethylated) Reverse | AAAAACATACCACCAAACCTAC |
| REC8 (methylated) Forward | GGATTATAGGCGCGTGTTATTAC |
| REC8 (methylated) Reverse | TAAAACCGAACGCAATAACTCAC |
| REC8 (unmethylated) Forward | TGGGATTATAGGTGTGTGTTATTAT |
| REC8 (unmethylated) Reverse | AATAAAACCAAACACAATAACTCAC |

Table 1

|  | Antibody | company name | catalog number | Dilution/amount |
| --- | --- | --- | --- | --- |
| Primary antibodies | BCL-2 | abcam | ab182858 | 1:2000 |
|  | Β-tubulin | abcam | ab179511 | 1:1000 |
|  | Cleaved PARP1 | abcam | ab32064 | 1:2000 |
|  | PARP1 | abcam | ab191217 | 1:1000 |
|  | Cleaved Caspase-3 | abcam | ab32042 | 1:500 |
|  | Caspase 3 | Proteintech | 19677-1-AP | 1:500 |
|  | gamma H2A.X | abcam | ab81299 | 1:5000 |
|  | XAF1 | abcam | ab17204 | 2ug/ml |
|  | ISG15 | Proteintech | 15981-1-AP | 1:1000 |
| Secondary antibodies | Goat Anti-Mouse | Epizyme | LF101 | 1:5000 |
|  | Goat Anti-Rabbit | Epizyme | LF102 | 1:5000 |
|  | Goat Anti-rabbit IgG Alexa Fluor 488 | abcam | ab150077 | 1:1000 |

Table2
